# Third generation EGFR TKIs: current data and future directions

**DOI:** 10.1186/s12943-018-0778-0

**Published:** 2018-02-19

**Authors:** Chee-Seng Tan, Nesaretnam Barr Kumarakulasinghe, Yi-Qing Huang, Yvonne Li En Ang, Joan Rou-En Choo, Boon-Cher Goh, Ross A. Soo

**Affiliations:** 1 Department of Haematology-Oncology, National University Cancer Institute of Singapore, National University Health System, 1E Kent Ridge Road, NUHS Tower Block, Level 7, Singapore, 119228 Singapore; 2 Cancer Science Institute of Singapore, National University of Singapore, Singapore, Singapore; 3 Department of Pharmacology, Yong Loo Lin School of Medicine, National University Health System, Singapore, Singapore; 4 School of Surgery, The University of Western Australia, Perth, Australia

**Keywords:** Third generation EGFR TKI, T790 M, Osimertinib, Resistance mechanism, FLAURA, Sequencing

## Abstract

Acquired *T790 M* mutation is the commonest cause of resistance for advanced non-small cell lung cancer (NSCLC) epidermal growth factor receptor (EGFR) mutant patients who had progressed after first line EGFR TKI (tyrosine kinase inhibitor). Several third generation EGFR TKIs which are EGFR mutant selective and wild-type (WT) sparing were developed to treat these patients with T790 M acquired resistant mutation. Osimertinib is one of the third generation EGFR TKIs and is currently the most advanced in clinical development. Unfortunately, despite good initial response, patients who was treated with third generation EGFR TKI would develop acquired resistance and several mechanisms had been identified and the commonest being C797S mutation at exon 20. Several novel treatment options were being developed for patients who had progressed on third generation EGFR TKI but they are still in the early phase of development. Osimertinib under FLAURA study had been shown to have better progression-free survival over first generation EGFR TKI in the first line setting and likely will become the new standard of care.

## Background

In 2009, the IPASS study established the superiority of gefitinib over chemotherapy for metastatic non-small cell lung cancer (NSCLC) patients with sensitizing epidermal growth factor receptor (EGFR) mutations [1]. Several first line phase III studies on first (gefitinib, erlotinib) and second (afatinib, dacomitinib) generation EGFR TKIs showed objective response rate and progression free survival (PFS) of patients with sensitizing *EGFR* to be 60–70% and 9 to 15 months, respectively [1–8].

Despite the initial high response rates, patients on EGFR TKIs will inevitably become resistant to treatment. Various mechanisms of acquired resistance have been identified and these can be divided into secondary mutations in EGFR, the activation of alternative signaling pathways, and phenotypic or histologic transformation [9–11]. The commonest mechanism of acquired resistance is T790 M mutation accounting for 50–60% of secondary resistance to primary EGFR TKI therapy [12]. This is also the basis for the development of third generation EGFR TKIs. The full discussion on the acquired mechanisms of resistance to first and second generation EGFR TKIs is beyond the scope of this article. Please refer to the following articles for a comprehensive review on this topic [9, 13].

### Third generation TKIs

Given the limited efficacy of second generation TKIs in circumventing T790 M resistance to first generation TKIs, third generation TKIs were developed. These include osimertinib, EGF816, olmutinib, PF-06747775, YH5448, avitinib and rociletinib. The defining characteristic of these third generation agents is that they have significantly greater activity in EGFR mutant cells than in EGFR WT cells, making them mutant-selective [14]. The only approved third generation TKI is osimertinib. In the rest of this article, we will review the preclinical and clinical data surrounding osimertinib and other third generation EGFR TKIs, as well as future challenges on the evaluation and treatment of resistance that arises from these third generation EGFR TKIs.

### Osimertinib: pre-clinical and clinical data

Osimertinib, an oral third-generation EGFR TKI selectively and irreversibly targets both sensitizing EGFR mutations as well as T790 M while sparing the wild-type EGFR tyrosine kinase [15]. Osimertinib, a mono-anilino-pyrimidine compound is less potent at inhibiting phosphorylation of EGFR in wild-type cell lines with close to 200 times greater potency against L858R/T790 M than wild-type EGFR [15]. In preclinical studies, osimertinib demonstrated impressive activity in xenograft and transgenic murine tumor models with both profound and sustained tumor regression [15]. In addition, osimertinib also induced sustained tumor regression in an EGFR-mutated mouse brain metastases model [16].

The Phase I/II AURA trial was conducted to determine the safety and efficacy of osimertinib in patients (*n* = 252) who progressed on initial EGFR TKIs [17]. Diarrhea was the most frequent toxicity (47%), followed by rash (40%), nausea and decreased appetite (21%). Despite G3 or higher toxicities noted in 32% of patients, only 7% and 6% of patients required a dose reduction or drug discontinuation. Of interest, 6 cases of potential pneumonitis-like events were reported. All 6 patients discontinued osimertinib. With regards to efficacy, the ORR was 51% and an impressive disease control rate (DCR) of 84%. And the median PFS was 8.2 months. As expected, the subgroup of T790 M-positive patients (*N* = 127) had an excellent DCR of 95%, ORR of 61% and median PFS of 9.6 months. Activity was lower in patients (*n* = 61) without *EGFR* T790 M mutations with an ORR and PFS of 21% and 2.8 months (95% confidence interval (CI) 2.1–4.3) respectively.

Following the encouraging efficacy and safety date from the initial AURA Phase I/II study, the single arm, multi-center phase II Aura 2 study was conducted with osimertinib at 80 mg orally daily [18]. All patients (*n* = 210) had advanced NSCLC harboring *EGFR* T790 M mutations that was centrally confirmed and had progressed on prior EGFR TKI therapy. The ORR was 70% with 3% complete responses and a DCR of 92%. The median PFS was 9.9 months (95% CI 8.5–12.3) with a median duration of response of 11.4 months. Overall, toxicities were manageable with the most common possibly treatment-related grade 3 or 4 AEs were prolonged electrocardiogram QT (2%), neutropenia (1%) and thrombocytopenia (1%).

In a pooled analysis of the AURA extension and AURA2 Phase II studies (*n* = 50), the central nervous system (CNS) ORR with osimertinib dose at 80 mg per day was 54% with 6 (12%) complete responders. 82% of patients responded intracranially by 6-week assessment [19].

AURA3 was an open-label, international, phase III trial of 419 patients with locally advanced or metastatic NSCLC with T790 M mutations randomized 2:1 to osimertinib at 80 mg daily (*n* = 279) or to standard-care pemetrexed plus platinum every 3 weeks, with maintenance pemetrexed allowed [20]. The median PFS was 10.1 months vs 4.4 months (hazard ratio (HR) 0.30, 95% CI 0.23–0.41, *p* < 0.001). A higher ORR was seen in the osimertinib arm when compared to standard of care: (71% vs 31%, odds ratio: 5.39, 95% CI 3.47–8.48, p < 0.001). The responses were also durable at 9.7 months in the osimertinib group compared with 4.1 months for chemotherapy. Furthermore, all the patient reported outcomes (PROs) were better in the osimertinib group than in the platinum-pemetrexed group.

In patients with CNS metastases, the median PFS was 8.5 months versus 4.2 months (HR 0.32, 95% CI 0.21–0.49). Only 5% (*n* = 13) vs 14% (*n* = 20) developed new CNS lesions while on treatment with osimertinib vs platinum/pemetrexed respectively. Among the patients receiving osimertinib, there was no significant difference in benefit between patients with T790 M-positive status on both tumor and plasma analyses and those in the intention-to-treat population.

Fewer patients reported adverse events of grade 3 or more in the osimertinib group (23%) than in the platinum–pemetrexed group (64%). In the osimertinib group, the most commonly reported adverse events were diarrhea (41%), rash (34%), dry skin (23%), and paronychia (22%). Interstitial lung disease–like adverse events were reported in 10 patients (4%) in the osimertinib group. Nine patients had grade ≤ 2 in severity and one death was reported. A prolongation in the QT interval was recorded in 10 patients (4%) in the osimertinib group and 1 patient (1%) in the platinum–pemetrexed group, with all events of grade 1 or 2 in severity except for one grade 3 event in the osimertinib group. Osimertinib was associated with a lower rate of permanent discontinuation, (7% compared with 10% with chemotherapy). Fatal adverse events were reported in 4 patients in the osimertinib group and one treatment related death in the platinum–pemetrexed group. Refer Table 1 for summarized clinical efficacy for osimertinib.

**Table 1:** Selected clinical efficacy on selected third generation EGFR TKIs in clinical development

Third Generation EGFR TKI	Status of development	Trial	Dose	n	ORR	PFS (mth) (95% CI)	Adverse events (selected all grade)
Osimertinib (AZD9291)	Approved for T790 M mutation post EGFR TKI	AURA 3 NCT01802632	80 mg daily	279	71% (65–76%)	10.1 (8.3–12.3)	Diarrhea (41%); rash (34%), paronychia (22%); pneumonitis (4%)
Olmutinib	Developing in Korea	NCT01588145	800 mg daily	76	56%	7.0 (5.5–8.3)	Diarrhea (55%), rash (39%), nauseas (38%).
Nazartinib (EGF816)	Phase I/II	NCT02108964	75-350 mg QD	132	44%	9.2 (9.0-NE)	Diarrhea (40%), maculopapular rash (39%), pruritus (32%), stomatitis (23%), and fatigue (21%).
Avitinib (AC0010)	Phase I/II (phase II AEGIS-1 trial had started)	NCT02330367	50-350 mg BID 150-300 mg cohort	136 95	44% ^a^ 51%	NA	Diarrhea (38%) and rash (24%)

*EGFR* epidermal growth factor receptor, *TKI* tyrosine kinase inhibitor, *ORR* objective response rate, *PFS* progression free survival, *n* number of participant, *NE* not evaluable, *NA* not available

^a^ including unconfirmed responses

In November 2015, osimertinib received accelerated approval under the Breakthrough Therapy Designation Program for metastatic epidermal growth factor receptor (EGFR) T790 M mutation-positive non-small cell lung cancer (NSCLC), as detected by an US FDA-approved test, whose disease has progressed on or after EGFR tyrosine kinase inhibitor (TKI) therapy. This was followed by recommendation by The European Medicines Agency (EMA) for conditional marketing authorization for Tagrisso (osimertinib) for same indication in December 2015 with marketing authorization approved in February 2016. Subsequently, Osimertinib received US FDA approval on March 30, 2017 based the confirmatory AURA3 study [20].

Osimertinib was evaluated in the front line setting compared to 1st generation EGFR TKIs in the FLAURA study. FLAURA was a Phase III, double-blind, randomized study assessing efficacy and safety of osimertinib versus standard of care EGFR-TKI (gefitinib or erlotinib) in the first-line treatment of patients (*n* = 556) with Ex19del/L858R EGFR mutated advanced NSCLC [21].

The primary endpoint PFS was 18.9 Months vs 10.2 months (HR 0.46, 0.37–0.57; *p* < 0.0001) and the PFS benefit was consistent across all subgroups. Of special interest, the PFS of patients with known brain metastasis at study entry treated with osimertinib (HR 0.47) was similar to patients without known brain metastasis (HR 0.46). CNS progression was also significantly lower in patients treated with osimertinib 6% vs 15%.

The ORR was similar for osimertinib and standard of care EGFR TKI at 80% and 76%, respectively. The median duration of response was significantly longer in patients treated with osimertinib (17.2 vs 8.5 months). The overall survival (OS) data was only 25% mature at time of analysis and was not statistically significant however did show a positive trend (HR 0.63, 0.45–0.88; *p* = 0.0068). A *p*-value of 0.0015 was required for statistical significance at the current OS maturity. Final OS analysis will be completed at approximately 60% maturity.

Compared to first generation EGFR TKI, osimertinib resulted in similar incidence of diarrhea (58% vs 57%), higher risk of stomatitis (29% vs 20%), lower incidence of dermatitis acneiform (25% vs 48%), elevated AST (9% VS 25%) and elevated ALT (8% VS 27%). Grade ≥ 3 adverse events occurred in 34% of osimertinib patients vs 45% in standard of care (SoC). Discontinuation of treatment due to adverse events occurred in 13% of osimertinib patients vs 18% receiving SoC.

Based on the results from the FLAURA study, osimertinib can be consider a standard of care for patients with metastatic NSCLC with EGFR sensitizing mutations especially in patients with brain metastasis.

### Other third generation EGFR TKI under development

**Fig. 1:**
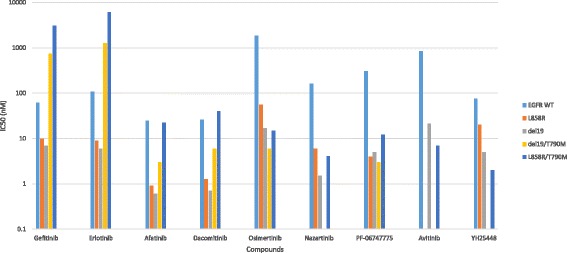
Pre -clinical efficacy based on IC50 (nM) comparing between first, second and selected third generation EGFR TKIs. EGFR WT = epidermal growth factor wild-type. EGFR WT is based on H2073 cell line for gefitinib, erlotinib, afatinib, dacomitinib, osimertinib; HaCaT cell line for nazartinib; A549 cell line for PF-06747775, A431 cell line for avitinib. L858R is based on H3255 cell line for all compounds. del19 is based on PC9 cell line for all compounds except HCC 827 cell line for nazartinib. del19/T790 M is based on PC9VanR cell line for all compounds. L858R/T790 M is based on H1975 cell line for all compounds

Refer to Table 1 for summarized clinical efficacy of selected third generation EGFR TKIs that are in clinical development. Figure 1 summarized the pre -clinical efficacy based on IC50 nanoMolar (nM) comparing between first, second and selected third generation EGFR TKIs.

### Olmutinib (BI 1482694/HM61713)

Olmutinib was investigated in phase I/II trial evaluating Korean NSCLC patients who had failed prior EGFR TKI. The recommended phase II dose was 800 mg daily. In the phase II study of patients who were T790 M and the ORR was 56% achieved ORR with DCR of 90%. The median PFS was 7.0 months (95% CI 5.5–8.3). The commonest all grade adverse events were diarrhea (55%), rash (39%), nauseas (38%) [22].

Boehringer Ingelheim collaborated with Hanmi Pharmaceutical to develop olmutinib in ELUXA trials. But the collaboration was halted in view of a South Korean Authority drug safety report of a fatal case of toxic epidermal necrolysis (TEN) [23, 24]. Olmutinib is currently being developed by Hanmi Pharmaceutical in South Korea.

### Nazartinib (EGF816)

Pre-clinical data showed that nazartinib showed similar mutant-selectivity and *EGFR* wild-type sparing property similar to other third generation EGFR TKIs [25].

In a phase I dose-escalation study of nazartinib (*n* = 132) over seven dose cohorts (75-350 mg QD), the confirmed ORR in 127 evaluable patients was 44% (56/127) with a disease control rate of 91%. The median PFS was 9.2 months (95% CI 9.0-NE) [26]. All grade adverse events included diarrhea (40%), maculopapular rash (39%), pruritus (32%), dry skin (23%), stomatitis (23%), and fatigue (21%). Grade 3 or 4 adverse events included maculopapular rash (14%), anemia (6%), and diarrhea (6%). Hepatitis B reactivation was reported in two patients who were not on antiviral prophylaxis. One patient resumed nazartinib after starting anti-viral and another patient died [26]. Nazartinib is also concurrently being investigated in combination with capmatinib (INC28), a c-MET inhibitor, in a phase I/II study (NCT02335944).

### PF-06747775

PF-06459988 is an irreversible pyrrolopyrimidine inhibitor of EGFR T790 M mutants. It has potent pre-clinical EGFR activity against the four common mutants (exon 19 deletion (Del), L858R, and double mutants T790 M/L858R and T790 M/Del), selectivity over wild-type EGFR [27].

In a phase I study where 44 EGFR mutant patients who had progressed on first line EGFR TKI were enrolled into six dose escalation (25 mg–600 mg) and 2 dose expansion cohorts (200 mg and 300 mg), the recommended phase 2 dose was 200 mg daily. All grade adverse events of > 25% included diarrhea (57%), rash (59%), paronychia (52%), dermatitis acneiform (34%), stomatitis (32%), pruritus (27%), dry skin (25%), and rhinorrhea (25%). Commonest grade 3 diarrhea and skin toxicity which were and easily managed. No grade 4 treatment related AEs were reported. Efficacy data is on-going [28].

### Avitinib (AC0010)

Avitinib is a pyrrolopyrimidine-based irreversible EGFR inhibitor and is structurally distinct from other pyrimidine-based irreversible EGFR inhibitors such as osimertinib and has activity against EGFR mutations including T790 M whilst spares EGFR WT [29].

Avitinib was investigated in a phase I/II study for EGFR mutant patients who had progressed on first line EGFR TKI [30]. A total of 136 patients were treated across seven dose cohorts (50-350 mg BID). Responses were observed in all dose cohorts except 50 mg BID. The ORR (including unconfirmed responses) and disease control rate (DCR) were 44% and 84% respectively. In the dose cohorts of 150-300 mg BID had ORR and DCR of 51% and 89% respectively. The recommended phase 2 dose (RP2D) was 300 mg BID. Avitinib was well tolerated with diarrhea (38%) and rash (24%) which was predominantly of grade 1 or 2 severity. Grade 3 or 4 side effects included diarrhea (2%) rash (2%) transaminitis (2–4%) [30]. In a subset of patients with brain metastases, the intracranial PFS of two patients were shorter than extracranial PFS. This finding may be attributed to a low blood-brain-barrier penetration rate of 0.046%–0.146% [31].

### YH25448

Early in vivo and in vitro data reported YH25448 had more potent inhibition of cancer cell growth compared to osimertinib in cancer cells harboring EGFR mutations (L858R/T790 M) with IC50 of 2 nM vs 8 nM and GI50 of 3.6 nM vs 11.8 nM respectively. YH25448 treatment in mice implanted with H1975 cells showed regression of tumor in both subcutaneous and intracranial lesions. At 10–25 mg/kg, YH25448 achieved more significant, complete tumor growth inhibition and longer overall survival compared to same doses of osimertinib [32]. Ongoing effort is underway to develop this compound further.

### Rociletinib (CO-1686)

Rociletinib was initially investigated in phase I/II study in TIGER-X trial for patients who had failed EGFR TKI in the first line setting. The ORR in patients harboring T790 M was 59% and the DCR was 93%. An updated pooled TIGER-X/TIGER-2 analysis reported a lower response rate of 34% for 625 mg b.i.d. dose cohort (*n* = 170) and 28% for 500 mg b.i.d. dose cohort (*n* = 79) [33]. In the latest analysis of TIGER-X reported in June 2016, the confirmed ORR was 45% (95% CI, 31 to 60) and 18 patients with T790 M-negative disease, the confirmed ORR was 17% (95% CI, 4 to 41) [34]. Based on this updated data showing lower than expected efficacy, FDA voted against the accelerated approval of rociletinib and Clovis subsequently stopped the clinical development.

### ASP8273

In a phase I/II trial of ASP8273 in Japanese patients with EGFR mutant NSCLC who had progressed on first line EGFR TKI, the ORR was 50%for all patients dosed with ≥100 mg with ORR of and 80% in T790 M positive patients. The most common all grade adverse events were diarrhea (56%), nausea (31%), vomiting (31%) and thrombocytopenia (31%). Less commonly, skin rash (9%) and interstitial lung disease (ILD)-like events (2%). Maximum tolerated dose (MTD) was determined at 400 mg and the R2PD dose was 300 mg [35].

In a North American study (*n* = 60), of patients with EGFR mutated NSCLC who have progressed after EGFR TKI90% of patients had T790 M mutation. In the T790 M positive cohort, the ORR was 37.5% (15/40) and DCR was 65% (26/40). The median PFS was 6.7 months (95% CI: 5.32–9.79 months) [36]. AA phase III study evaluating ASP8273 versus first generation EGFR TKI in the first line treatment of EGFR mutant advanced NSCLC (SOLAR) was subsequently initiated. However, on the recommendation of the Independent Data Monitoring Committee, the trial was discontinued in May 2017 due to lack of clinical efficacy [37].

### Challenges in treatment of advanced EGFR mutant patients

- Detection of EGFR T790 M mutations with plasma sample

At disease progression, a biopsy of tumor tissue is typically performed to evaluate for targetable resistance mechanisms such as EGFR T790 M mutation. However, procedures are invasive and not always feasible in patients with advanced disease, with up to 28% of NSCLC patients unable to provide a biopsy sample suitable for mutational analysis [38]. “Liquid biopsy” has been developed to counter conventional tissue biopsy limitations. Benefits over tissue biopsy include the ability to capture tumor heterogeneity and to quantify the number of mutated gene copies which is useful for monitoring disease response and predicting early treatment failure [39, 40]. Importantly, it is noninvasive, where repeated testing can be done for dynamic monitoring of tumor molecular changes [41].

### Circulating tumor cells (CTCs)

Once isolated, CTCs can be used to analyze EGFR mutational status [42]. Additionally, immunohistochemistry or fluorescence in-situ hybridization which cannot be done on fragmented plasma ctDNA samples, can be performed on CTCs to evaluate for other rearrangements, mutations or amplifications [43].

### Plasma cell-free circulating tumor DNA (ctDNA) for the detection of T790 M

Several platforms have been used to detect ctDNA and these included the amplified refractory mutation system (ARMS), peptide nucleic acid mediated polymerase chain reaction (PCR) clamping, digital PCR, denaturing high performance liquid chromatography (DHPLC) and next generation sequencing (NGS). A comprehensive review of “liquid biopsy” is beyond the scope of this review article. Please refer to these review articles for more information [44, 45].

Of the methods available, ARMS, ddPCR and BEAMing have been determined to be clinically applicable in the setting of resistance to EGFR TKIs and the development of the T790 M mutation. Studies have demonstrated high sensitivity, specificity and good concordance between these strategies and actual tissue biopsy results. Thress and colleagues evaluated plasma samples from patients recruited to the AURA 1 trial prior to initiation of osimertinib. The sensitivity and specificity were 73% and 67% respectively with cobas®, and 81% and 58% respectively with BEAMing in detection of T790 M. Concordance between the platforms was > 90% [46]. Karlovich et al. also demonstrated a high concordance rate between matched plasma and tumor tissue samples drawn from the phase I clinical trial of rociletinib, where the concordance rate for T790 M was 64% with cobas® and 73% with BEAMing [47]. Table 2 summarizes the sensitivity and specificity for T790 M mutation testing based on various plasma assay platforms.

b) Mechanisms of resistance to 3rd generation TKI

**Table 2:** Summary of the sensitivity and specificity for T790 M mutation testing based on various plasma assay platforms

Plasma Assays		Sensitivity n/N (%)		Specificity n/N (%)		Plasma T790 M positive / histology negative (%)	Reference
		EGFR	T790 M after PD on TKI	EGFR	T790 M after PD on TKI		
Digital PCR, Microdroplet	BEAMing	49/60 (81.7)	33/45 (73.3)	2/3 (66.7)	9/18 (50)	9/18 (50)	Karlovich 2016 (Roci)
		43/51 (84.3)	33/41 (80.5)	65/67 (97.0)	14/24 (58.3)	10/24 (41.7)	Thress 2015 (AURA)
		112/136 (82.3)	111/158 (70.3)	78/80 (97.5)	40/58 (69.0)	18/40 (31.0)	Oxnard 2016
	ddPCR	NA	6/9 (66.7)	NA	5/7 (71.4)	2/7 (28.6)	Wang 2017
		25/33 (75.7)	20/31 (64.5)	7/8 (87.5)	7/10 (70)	3/10 (30)	Takahama 2016 (West Japan Oncology Group)
		9/10 (90)	12/17 (70.5)	28/28 (100)	5/6 (83.3)	1/6 (16.7)	Thress 2015 (AURA)
Allele -specific PCR	ARMS, Therascreen	30/37 (81.1)	5/17 (29.4)	38/38 (100)	6/6 (100)	0	Thress 2015 (AURA)
	Roche cobas® AS-PCR	43/51 (84.3)	30/41 (73.2)	65/67 (97.0)	16/24 (66.7)	8/24 (33.3)	Thress 2015 (AURA)
		55/75 (73.3)	21/33 (63.6)	24/24 (100)	61/62 (98.4)	1/62 (1.6)	Karlovich et al. 2016 (Roci)
		NA	254/414 (61.3)	NA	99/126 (78.6)	27/126 (21.4)	Jenkins 2017 (AURA ext. + AURA2 pooled), excluded those with unknown plasma results
		13/16 (81.2)	6/10 (60.0)	30/30 (100)	9/15 (60)	6/15 (40)	Sundaresan 2016
CTCs		NA	5/9 (55.5)	NA	7/12 (58.2)	5/12 (41.7)	Sundaresan 2016

Despite initial impressive outcomes with 3rd generation EGFR TKIs, acquired resistance invariably develops. Several mechanisms of resistance that are EGFR-dependent and EGFR-independent have been described. EGFR-dependent mechanisms include the development of *EGFR* C797S mutation whereas examples of EGFR-independent mechanisms include activation of pathways downstream of EGFR and parallel signaling pathways (Table 3).

**Table 3:** Mechanisms of resistance to third generation EGFR TKIs

Mechanism	Author	3rd gen EGFR TKI	Sample	Method	Other mechanisms (no. of patients)
EGFR-dependent mechanisms of resistance to third generation TKIs					
C797S	Thress et al. Yu et al. Ortiz Cuaran Ramalingam Ig Ou Ou ASCO Piotrowska WCLC	Osimertinib	Plasma/Tissue Tissue Tissue Plasma Plasma Plasma/Tissue Plasma	NGS,ddPCR NGS NGS NGS NGS NGS NGS	-- -- MET Amp P53 (1) G796, L792 L792(8), L718 (1), G796(1), PIK3CA (4) EGFR Amp (29), MET Amp (10), BRAF (3), PIK3CA (9)
	Tan ASCO	Nazartinib	Tissue	NGS	MTOR del
	Chabon	Rociletinib	Plasma	CAPP-Seq	–
	Song	Olmutinib	Tissue	NGS	–
C797G	Menon	Osimertinib	Tissue	NGS	EGFR and MYC Amp
L792	Ou ASCO Chen Ig Ou	Osimertinib	Plasma/Tissue Plasma Plasma	NGS NGS NGS	C797S (8), PIK3CA (2) C797S (3) C797S, G796
L718	Ou ASCO Bersanelli	Osimertinib	Plasma/Tissue Tissue	NGS NGS	C797S (1), L792(2), G796(1), KRAS(1) --
G796	Ig Ou Ou ASCO	Osimertinib	Plasma Plasma/Tissue	NGS NGS	L792, C797S L792, C797S (1), L718(1)
L798	Chabon	Rociletinib	Plasma	CAPP-Seq	EGFR Amp
E709K	Chabon	Rociletinib	Plasma	CAPP-Seq	–
L692 V	Chabon	Rociletinib	Plasma	CAPP-Seq	–
EGFR-independent mechanisms of resistance to third generation TKIs					
Her 2 Amp	Planchard Oxnard Ramalingam	Osimertinib	Tissue Plasma/Tissue Plasma	CGH/FISH NGS/CGH NGS	-- -- P53, IDH2
	Ortiz-Cuaran	Rociletinib/ Osimertinib	Tissue	FISH	MET Amp(1)
	Chabon	Rociletinib	Plasma	CAPP-Seq	MET Amp(1), CDKN2A(1), EGFR Amp and PIK3CA(1)
MET Amp	Planchard Ou Chia Ramalingam Ortiz-Cuaran Piotrowska ASCO	Osimertinib	Tissue Tissue Tissue Plasma Tissue Plasma/Tissue	NGS/CGH/IHC ddPCR NGS FISH FISH	-- -- -- RB1 mut, P53 Her2 Amp (1) --
	Tan ASCO	Nazartinib	Tissue	NGS	BRAF fusions
	Chabon	Rociletinib	Plasma	CAPP-Seq	CDKN2A(1), PIK3CA(1), PIK3CA, KRAS and MET Amp(1),Her2 Amp(1)
KRAS mut	Ramalingam Ortiz-Cuaran	Osimertinib	Plasma Tissue	NGS NGS	CTNNB1 C797S plasma
	Chabon	Rociletinib	Plasma	CAPP-Seq	MET mut, MET amp, PIK3CA(1), KIT mut(1)
NRAS mut	Eberlein	Osimertinib	Cell lines	NGS	–
BRAF mut	Oxnard Ho et al.	Osimertinib	Tissue Cell lines	NGS MALDI	-- --
PIK3CA mut	Oxnard Ramalingam	Osimertinib	Tissue Plasma	NGS NGS	-- P53, PTEN mut, NOTCH
	Chabon	Rociletinib	Plasma	CAPP-Seq	MET Amp (1), MET Amp, MET mut, KRAS(1), EGFR and Her 2 Amp(1)
PTEN loss	Kim	Osimertinib	Tissue	NGS	–
FGF2-FGFR1 Autocrine loop	Kim Piotrowska ASCO	Osimertinib	Tissue Plasma/Tissue	NGS NGS	-- --
SCLC	Kim Ham Li	Osimertinib	Tissue Tissue Tissue/Plasma	NGS NGS NGS	RB1 loss EGFR Amp(1) P53, PTEN, PIK3CA
	Piotrowska	Rociletinib	Tissue/Plasma	NGS	RB1 loss(1), RB1 mut(1)

*Amp* amplification, *BRAF* B-Raf proto-oncogene, *CAPP-Seq* cancer personal profiling by deep sequencing, *CDKN2A* cyclin dependent kinase inhibitor 2A, *CGH* comparative genomic hybridization, *CTNNB1* catenin beta 1 gene, *ddPCR* droplet digital polymerase chain reaction, *EGFR* Epidermal growth factor receptor, *FGF2-FGFR1* FGF2-fibroblast growth factor receptor 1 (FGFR1), *FISH* fluorescent in situ hybridization, *Her2* erb-b2 receptor tyrosine kinase 2, *IDH2* isocitrate dehydrogenase 2, *IHC* immunohistochemistry, *KIT* KIT proto-oncogene receptor tyrosine kinase, *KRAS* KRAS proto-oncogene, *MALDI* matrix assisted laser desorption ionization–time of flight mass, *MET* MET proto-oncogene, *MTOR* mechanistic target of rapamysin kinase, *mut* mutation, *MYC* MYC proto-oncogene, *NGS* next generation sequencing, *NOTCH* NOTCH gene, *NRAS* NRAS proto-oncogene, *PIK3CA* phosphatidylinositol-4,5-bisphosphate 3-kinase catalytic subunit alpha, *PTEN* Phosphatase and tensin homolog, *P53* tumour protein P53, *RB1* RB transcriptional corepressor 1, *SCLC* small cell lung cancer, *3rd gen TKI* third-generation tyrosine kinase inhibitor

### EGFR C797 mutation

One of the first mutations reported was the C797S mutation, a point mutation on exon 20. This mutation abolishes the covalent bonding of osimertinib to EGFR [48]. Its prevalence ranges between 22% to 40%-- being identified in 22 out of 99 NSCLC patients who have progressed on osimertinib [49], and 6 out of 15 patients in the phase I AURA study [50] respectively.

In addition to osimertinib, C797S mutation has also been reported to mediate resistance to other third-generation TKIs, such as HM61713 olmutinib [51], rociletinib [52] and nazartinib [53]. Chabon and colleagues analyzed pre- and post- treatment samples from 43 patients treated with rociletinib. Only 1 out of 43 (2%) developed C797S mutation *in cis* with T790 M, a frequency that is lower compared to that of Osimertinib. Piotrowska also found no C797S mutation in a group of 12 patients who progressed on rociletinib [54]. These evidences suggest a potential difference in the pattern of resistance between osimertinib and rociletinib. Recently, Tan et al. reported genomic profile of 9 resistant tumor samples, following progression on EGF816 nazartinib. C797S mutation was identified in one patient, who also had concurrent MTOR deletion [53].

Preclinical studies have demonstrated the acquired C797S mutation in cells resistant to 3rd generation TKIs [48, 55]. Of interest was the finding that the allelic context in which C797S was acquired may predict responsiveness to subsequent TKI treatments [55].

A recent study by Piotrowska and colleagues evaluated Guardant Health database of plasma samples of 61 lung adenocarcinoma patients with C797S mutation. These patients had acquired T790 M mutation and were treated with osimertinib. The study found the following C797S configuration: C797S/T790 M *in cis* in 50 patients (82%); C797S/T790 M *in trans* in 6 patients (10%); C797S alone without T790 M in 4 patients (6%); and 1 patient (2%) had two co-existent C797S clones (one *in cis* with T790 M and one *in trans*) [56]. In addition, 51 patients (84%) had at least one bona fide resistance mechanism co-occurring with C797S, namely *EGFR* amplification (*n* = 29; 48%); *MET* amplification (*n* = 10; 16%); *BRAF* V600E (*n* = 3; 5%) and *PIK3CA* mutation (*n* = 9; 15%). Interestingly, C797S can sometimes be polyclonal within individual patients. Thus, the polyclonality of C797S, together with co-existing resistance mechanisms, highlight the heterogeneity of resistant EGFR-mutant cancers.

Other than C797S, a case report by Menon et al. demonstrated a novel C797 variant in a patient who has progressed on osimertinib. The authors found a C797G mutation *in cis* with T790 M. Focal *MYC* and *EGFR* amplifications were also isolated in the same patient [57].

### Other EGFR mutations

In addition to the C797S mutation, other *EGFR* mutations such as L792 and L718 mutations have also been reported [58–61]. Interestingly, all L792 mutations are *in cis* with T790 M and *in trans* with C797 mutations when present in the same patient. In addition, 2 out of 10 L792-positive patients and 6 out of 7 L718-positive patients did not have co-existing C797 mutations. This suggests that C797-, L792- and L718- mutated cells are likely different resistant clones [58].

Other *EGFR* L798I, E709K, L792 V and G796S/R mutations had also been described [52, 59, 62, 63].

### Bypass mechanisms

Other resistance mechanisms to osimertinib identified involve either activation of pathways downstream of EGFR (RAS-MAPK pathway signaling) or those that activate parallel signaling pathways, such as *Her2* amplification, *MET* amplification, *PTEN* loss and *PIK3CA* mutation.

#### RAS-MAPK

*KRAS* mutation, *KRAS* amplification, *BRAF*, *NRAS* (including a novel *NRAS* E6K mutation) and *MEK1* mutation have been described as mechanisms of acquired resistance to third generation TKIs [49, 52, 64–66]. *KRAS* G12S, G12A, Q61H, A146T and G12D mutation had been reported post third generation EGFR TKI [52, 64, 65].

Other than *KRAS* mutations, *NRAS* mutations have also been reported preclinically. *NRAS* missense mutations (including a novel E63K mutation) or NRAS copy number gain had been reported post osimertinib. Interestingly, these resistant cell lines were sensitive to combination therapy of *MEK* inhibitor selumetinib with EGFR TKI [66].

Lastly, a *BRAF* V600E mutation was uncovered as resistant mechanism to osimertinib in two reports [49, 67]. Notably, in one study, combination of BRAF inhibitor encorafenib together with osimertinib was attempted and led to significant inhibitory effects on cell lines [67].

#### *Her2* and *MET* amplification

Amplification of *Her2* and *MET* have been described after progression on third generation TKIs [49, 52, 53, 64, 65, 68–70]. Interestingly, *Her2* amplification and T790 M mutation appear to be mutually exclusive in patients who progressed on osimertinib [49, 64, 68] but may co-exist in patients who progressed on rociletinib [52].

*MET* amplification has been described in both pre-clinical and clinical studies. Preclinical studies had shown *MET* amplification as a resistance mechanism to third generation TKI [71]. *MET* amplification had also been reported for patient who progressed on osimertinib [69, 70], rociletinib [52] and nazartinib. [53]

### PIK3CA mutations

*PIK3CA* E545K mutation has been described as a resistant mechanism to osimertinib in at least two reports [49, 64]. Two *PIK3CA* gene mutations (E545K, E542K) were also described in 5 out of 43 patients who developed resistance to rociletinib [52].

### FGF2-fibroblast growth factor receptor (FGFR1)

In vitro analysis demonstrated that FGF2 supplement conferred resistance to osimertinib in EGFR-mutant NSCLC cells [72]. Clinically *FGFR* amplification after progression on osimertinib were reported after osimertinib [72].

### Small cell transformation

Small cell lung cancer (SCLC) transformation- a known rare mechanism of resistance to first generation TKI, has been described after treatment with third generation TKIs [54, 72–74]. These transformed SCLCs can continue to harbor their original EGFR-activating mutations, but not T790 M [54, 72–74]. Genome sequencing revealed *RB1* mutation and loss of *RB1* in these SCLC after acquired resistance to 3rd generation TKI, suggesting that these mutations play critical roles in driving the transformation [54, 72]. Co-occurring P53, PTEN and PIK3CA mutations have also been reported in a patient with small cell transformation after osimertinib [74].

### Future directions

- Overcoming acquired resistance to third generation EGFR TKI

### Fourth generation EGFR TKIs

The fourth generation EGFR TKIs are also under development. The current EGFR TKIs all target the ATP-binding site; however, the C797S mutation blocks the covalent binding of these drugs, conferring resistance. EAI001 and EAI045 were rationally identified as a molecule that binds allosterically to EGFR away from the binding site (non-ATP competitive), with specificity for mutant EGFR over wildtype EGFR [75]. EAI001 was found to have activity against L858R/T790 M mutant EGFR, but was less active against individual L858R or T790 M mutant EGFR. EAI045 was active in cell lines with individual L858R or T790 M mutations, or both [76]. In Ba/F3 cell lines bearing L858R/T790 M/C797S mutations, EAI045 was shown to result in control of cell proliferation when used in combination with cetuximab, but not when used as a single agent. This was attributed to the fact that if an EGFR dimer contained a wild-type and a mutant EGFR molecule, there would be differing susceptibilities to EAI045, compromising its activity. With the anti-EGFR monoclonal antibody cetuximab blocking EGFR dimerization, EAI045 can block these molecules in a monomer state [76]. Similar results were seen in mouse models carrying L8585R/T790 M/C797S [76, 77].

### Other novel EGFR inhibitors

Gunther and colleagues recently developed a new class of trisubstituted pyridinyl imidazole EGFR inhibitors based on a p38 MAP kinase inhibitor compound [78, 79]. Using molecular modeling, authors synthesized 40 compounds with activity against EGFR mutants and systematically developed metabolically stable noncovalent reversible EGFR inhibitors. These compounds demonstrated efficacy against cells expressing the triple mutation (T790 M/C797S/L858R) with IC50 values of less than 10 nM and also had more than 300-fold selectivity for double EGFR mutant (T790 M/L858R) cells over wild-type EGFR. Further studies need to be done to evaluate the clinical efficacy and safety of these new compounds.

### First generation EGFR TKIs after acquired resistance to third generation TKIs

Niederst et al. further described that cell lines harboring dual C797S and EGFR activating mutations (C797S/del19) without the T790 M mutation were resistant to third-generation TKIs but retained sensitivity to gefitinib or afatinib [55]. In a patient who progressed on osimertinib and developed the dual EGFR mutation (C797S/del19), treatment with gefinitib resulted in partial response and meaningful clinical improvement [80]. This suggests that patients treated with third generation TKIs in the first line setting who acquire resistance driven by C797S but remain undetectable for T790 M may subsequently respond to first-generation TKIs.

### First and third generation EGFR TKI combinations

The configuration of T790 M and C797S mutations using cell line MGH121 Res #1 has been found to be an important feature in predicting response to treatment. When the mutations occur in trans (i.e. on separate alleles), cells are resistant to third generation EGFR TKIs but sensitive to a combination of first and third generation EGFR TKIs. However, when the mutations occur in cis, no EGFR TKIs alone or in combination are effective. [55] In a patient who developed the triple mutation (T790 M/C797S/del19) in trans after progression on osimertinib, the combination of erlotinib and osimertinib was able to achieve partial response with undetectable C797S by ctDNA analysis after one month and further sustained response after two months of treatment. Strikingly, at disease progression after three months of therapy, C797S located in trans to T790 M remained undetectable but C797S in cis to T790 M appeared. The patient did not respond to further treatment with EGFR TKIs and subsequently required chemotherapy for disease control [81].

### Brigatinib and anti-EGFR antibodies

Brigatinib (AP-26113) is a dual anaplastic lymphoma kinase (ALK) and EGFR inhibitor. Uchibori and colleagues screened the growth inhibitory activity of 30 existing tyrosine kinase inhibitors against Ba/F3 cell lines overexpressing the triple mutation (T790 M/C797S/del19). They subsequently identified brigatinib as the only compound to have significant albeit modest activity in vitro and in vivo. Docking and molecular dynamic simulations demonstrated that brigatinib was able to bind to the triple-mutant EGFR ATP-binding pocket. Of interest, when combined with an anti-EGFR antibody (either cetuximab or panitumumab), there was enhanced efficacy against triple-mutant Ba/F3 cell lines, with a three-fold decrease in IC50 of brigatinib. This combination also successfully prolonged the survival of PC9 triple-mutant xenograft-bearing mice, at low toxicities. Brigatinib in combination with anti-EGFR antibodies is a promising strategy to overcoming triple mutations [82].

### Third generation TKIs in combination with MEK inhibitors

In vitro studies using PC9 cell lines harboring the triple mutation (T790 M/C797S/del19) revealed that modulation of Bim and Mcl-1 levels was critical to mediating resistance against osimertinib induced apoptosis. Use of a MEK inhibitor to suppress ERK-dependent phosphorylation of Bim and Mcl-1 restored the ability of osimertinib to induce apoptosis in these cells. These findings suggest co-targeting MEK/ERK signaling is another possible strategy to overcoming triple mutations [83].

### In combination with oxidative phosphorylation inhibitors

Use of osimertinib in combination with oxidative phosphorylation (OxPhos) inhibitors is another strategy currently being explored in the preclinical setting. Martin et al. demonstrated that EGFR mutant cell lines treated with osimertinib resulted in inhibition of glycolysis and consequent dependence on mitochondrial oxidative phosphorylation. Simultaneous treatment with OxPhos inhibitors (including phenformin, buformin, metformin, BAY 87–2243 and oligomycin) increased the sensitivity of EGFR mutant cells to osimertinib and was able to delay the development of osimertinib resistance in a dose dependent manner [84]. This represents a novel strategy that warrants further investigation.

### Osimertinib to treat EGF816 failure

A final strategy in the management of resistance to third generation TKIs is the use of alternative third generation TKIs. Cross-resistance between various third generation TKIs has not yet been well studied. A recent abstract published at ASCO 2017 recruited patients with T790 M mutations who had progressed on EGF816 and were subsequently given osimertinib. Osimertinib had a response rate of 14%, with a median treatment duration of 9 months, signifying meaningful clinical benefit [85]. This highlights the possibility of sequential third generation TKIs in the treatment of NSCLC.

b) Sequencing of EGFR TKIs:

Osimertinib has demonstrated better PFS and less toxicity compared to first generation EGFR TKI based on FLAURA results. Furthermore, it has been shown to be also effective for patients with CNS metastases. OS is immature at the present moment but unless it is detrimental, osimertinib will likely become the preferred EGFR TKI in the first line setting [21].

**Fig. 2:**
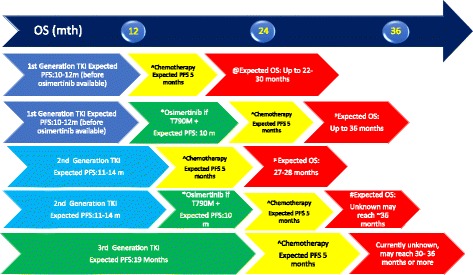
Potential Sequencing of EGFR Tyrosine Kinase Inhibitors and its Estimated Overall Survival (OS). @ Estimated based on First Line EGFR TKI studies IPASS, WJTOG3405. *Estimated based on Pooled analysis AURA Extension & AURA2 as well as AURA3 Study. P Estimated based on OS reported from the Pooled analysis AURA. Extension & AURA2 Reported OS: 26.8 months + 10–12 months expected PFS from 1st Gen TKI. μ updated OS from Lux Lung 7. #Currently limited data. Only ~ 10% of patients received osimertinib post progression on Afatinib in Lux Lung 7. OS for these 10% patients is not available. ^ Estimated based on AURA3

At present, there is no head-to-head comparison between osimertinib and second generation EGFR TKIs. Osimertinib in the first line setting demonstrated median PFS of around 19 months [21] as compared to second generation EGFR TKIs of around 11–14 months [7, 8]. Furthermore, the toxicity profiles of osimertinib which is WT sparing is much more favourable compared to second generation EGFR TKI which has higher rate of skin and diarrhea toxicities. See Fig. 2 for illustration of overall survival estimation from various sequencing potential of EGFR TKIs.

Only about half of patients who were started upfront with first or second generation EGFR TKI will develop T790 M acquired mutation which will allow the subsequent treatment with osimertinib. The rest of the patients would unfortunately be treated with conventional chemotherapy.

The acquired resistance mechanisms with upfront treatment with osimertinib is not well understood. These patients at present do not have any clear options aside from cytotoxic chemotherapy or enrolment into a clinical trial. Early report from phase I AURA study, 3 out of 9 patients had both p53 and RB1 mutations which are known to be predictive of small cell transformation [64]. The dataset is very small at the present moment and should be interpreted with caution.

## Abbreviations

ARMS: amplified refractory mutation system
CI: confidence interval
CNS: central nervous system
CTCs: circulating tumor cells
ctDNA: circulating tumor DNA
DCR: disease control rate
DHPLC: denaturing high performance liquid chromatography
EGFR: epidermal growth factor receptor
FGFR: fibroblast growth factor receptor
HR: hazard ratio
ILD: interstitial lung disease
MTD: maximum tolerated dose
NGS: next generation sequencing
nM: nanomolar
NSCLC: non-small cell lung cancer
ORR: objective response rate
PCR: polymerase chain reaction
PFS: progression free survival
PROs: patient reported outcomes
RP2D: recommended phase II dose
SoC: standard of care
TEN: toxic epidermal necrolysis
TKI: tyrosine kinase inhibitor
WT: wild-type
